# Gender in the allocation of organs in kidney transplants: meta-analysis

**DOI:** 10.1590/S0034-8910.2015049005822

**Published:** 2015-09-23

**Authors:** Erika Vieira Almeida e Santiago, Micheline Rosa Silveira, Vânia Eloisa de Araújo, Katia de Paula Farah, Francisco de Assis Acurcio, Maria das Graças Braga Ceccato

**Affiliations:** I Programa de Pós-Graduação em Medicamentos e Assistência Farmacêutica. Faculdade de Farmácia. Universidade Federal de Minas Gerais. Belo Horizonte, MG, Brasil; II Departamento de Odontologia. Instituto de Ciências Biológicas e da Saúde. Pontifícia Universidade Católica de Minas Gerais. Belo Horizonte, MG, Brasil; III Departamento de Clínica Médica. Faculdade de Medicina. Universidade Federal de Minas Gerais. Belo Horizonte, MG, Brasil

**Keywords:** Kidney Transplantation, Sex Distribution, Gender and Health, Prognosis, Meta-Analysis

## Abstract

**OBJECTIVE:**

To analyze whether gender influence survival results of kidney transplant grafts and patients.

**METHODS:**

Systematic review with meta-analysis of cohort studies available on Medline (PubMed), LILACS, CENTRAL, and Embase databases, including manual searching and in the grey literature. The selection of studies and the collection of data were conducted twice by independent reviewers, and disagreements were settled by a third reviewer. Graft and patient survival rates were evaluated as effectiveness measurements. Meta-analysis was conducted with the Review Manager^®^ 5.2 software, through the application of a random effects model. Recipient, donor, and donor-recipient gender comparisons were evaluated.

**RESULTS:**

: Twenty-nine studies involving 765,753 patients were included. Regarding graft survival, those from male donors were observed to have longer survival rates as compared to the ones from female donors, only regarding a 10-year follow-up period. Comparison between recipient genders was not found to have significant differences on any evaluated follow-up periods. In the evaluation between donor-recipient genders, male donor-male recipient transplants were favored in a statistically significant way. No statistically significant differences were observed in regards to patient survival for gender comparisons in all follow-up periods evaluated.

**CONCLUSIONS:**

The quantitative analysis of the studies suggests that donor or recipient genders, when evaluated isolatedly, do not influence patient or graft survival rates. However, the combination between donor-recipient genders may be a determining factor for graft survival.

## INTRODUCTION

Kidney transplants are considered to be the best therapeutic alternatives for persons suffering from advanced chronic kidney disease.^13^ ^,^ ^18^ ^,^ ^32^ Gender differences regarding kidney transplants have been reported in the literature and observed in the clinical practice over the last decades. They affect transplant results, such as in acute and chronic rejections and graft and patient survival rates. Women have less access to transplants. They have increased risk of acute rejection and decreased risk of chronic rejection – those risks increase with age.^9^ ^,^ ^22^ In turn, women are observed to account for around 65.0% of living kidney donors.^7^ ^,^ ^37^ The etiology of those differences is still unknown, but it probably reflects hormone, immunological, and aging differences, as well as prejudice.^7^ ^,^ ^22^ ^,^ ^28^

Survival rates are higher among women following kidney transplants,^9^ ^,^ ^11^ but the data are not confirmed by the literature. In a South African study, worse survival rates have been observed among women, but no significant differences were found between genders in graft survival.^23^ In another study, no differences were observed between genders in patient and graft survival rates.^27^

Knowing differences across genders is necessary to identify possible barriers in the achievement of ideal results and in the development of interventions that overcome those barriers. This review, by focusing on those gender-related differences in the clinical effectiveness of immunosuppressive therapies for kidney transplant maintenance, may promote better understanding, provide more efficient health care, contribute to the creation of clinical protocols, and promote better long-term results for patients.

The objective of this review was to analyze whether genders influence patient and graft survival rates in kidney transplants.

## METHODS

This review was conducted according to the recommendations from the Cochrane Collaboration Handbook.^a^ The article was prepared according to Preferred Reporting Items for Systematic reviews and Meta-Analysis (PRISMA).^21^

Observational cohort studies were selected. The selection included studies with patients who received kidney transplants from living or deceased donors for the first time or more than once, mentioning gender differences concerning pre-transplant characteristics, and finding survival results for grafts, patients, or for both. Studies that did not involve immunosuppressants for maintenance of kidney transplants, pharmacokinetic studies, economic evaluation studies, review studies, and studies conducted on animals were excluded, as per the exclusion criteria.

An electronic search was performed for articles published until December 2013, on Medline (PubMed), Latin-American and Caribbean Center on Health Sciences Information (LILACS), Cochrane Controlled Trials Databases (CENTRAL), Embase databases. Manual searches were also conducted in the reference lists of all studies selected from the published systematic review.^44^ Studies from the grey literature were also sought after: in the thesis and essay database from *Coordenação de Aperfeiçoamento de Pessoal de Nível Superior *(CAPES – Coordination for the Improvement of Undergraduate Personnel), in *Biblioteca Digital Brasileira de Teses e Dissertações* (Brazilian Digital Library of Thesis and Dissertations), and in Universidade de São Paulo’s Digital Library of Theses and Dissertations. There were no restrictions regarding dates and languages of publications. Table 1 describes the search strategy used in each surveyed database.

**Table 1:** Bibliographical search strategies for observational studies conducted in each database, on 12/12/2013.

Database	Studies	Search strategy
Medline (via PubMed)	3,263	((((((((((((((Transplantation, Kidney) OR Kidney Transplantations) OR Transplantations, Kidney) OR Transplantation, Renal) OR Renal Transplantation) OR Renal Transplantations) OR Transplantations, Renal) OR Grafting, Kidney) OR Kidney Grafting)) OR (Kidney Transplantation) OR (“Kidney Transplantation”[Mesh) AND (((((male[Title/Abstract) OR female[Title/Abstract) OR gender[Title/Abstract)) OR (((((((((Factor, Sex) OR Factors, Sex) OR Sex Factor)) OR (Sex Factors)) OR (“Sex Factors”[Mesh)) OR (((((((Characteristic, Sex) OR Characteristics, Sex) OR Sex Characteristic) OR Sex Differences) OR Difference, Sex) OR Differences, Sex) OR Sex Difference)) OR (Sex Characteristics)) OR (“Sex Characteristics”[Mesh))) AND Humans[Mesh)) AND ((((“Cohort Studies”[Mesh) OR (cohort study) OR (studies, cohort) OR (study, cohort) OR (concurrent studies) OR (studies, concurrent) OR (concurrent study) OR (study, concurrent) OR (historical cohort studies) OR (studies, historical cohort) OR (cohort studies, historical) OR (cohort study, historical) OR (historical cohort study) OR (study, historical cohort) OR (analysis, cohort) OR (analysis, cohort) OR (cohort analyses) OR (cohort analysis) OR (closed cohort studies) OR (cohort studies, closed) OR (closed cohort study) OR (cohort study, closed) OR (study, closed cohort) OR (studies, closed cohort) OR (incidence studies) OR (incidence study) OR (studies, incidence) OR (study, incidence) OR (cohort studies) OR (cohort) OR (cohort analysis) OR (cohort study) OR (prospective cohort) OR (retrospective cohort) OR (retrospective cohort study) OR (prospective cohort study) OR (“Follow-Up Studies”[Mesh) OR (follow up studies) OR (follow-up study) OR (studies, follow-up) OR (study, follow-up) OR followup studies OR (followup study) OR (studies, followup) OR (study, followup) OR (“Epidemiologic Studies”[Mesh OR “Cross-Sectional Studies”[Mesh OR “Retrospective Studies”[Mesh OR “Longitudinal Studies”[Mesh OR “Prospective Studies”[Mesh))) OR ((case* AND and control*[Text Word))))
Embase	2,363	‘kidney transplantation’/exp ANDembase/lim AND ‘gender and sex’/exp ANDembase/lim OR ‘sex difference’/exp ANDembase/lim OR ‘gender’/exp ANDembase/lim OR ‘sex ratio’/exp ANDembase/lim AND (‘cohort analysis’/de OR ‘comparative study’/de OR ‘control group’/de OR ‘controlled study’/de OR ‘human’/de OR ‘observational study’/de OR ‘outcomes research’/de OR ‘prospective study’/de OR ‘retrospective study’/de)
CENTRAL	280	(“Transplantation, Kidney” OR “Kidney Transplantations” OR “Transplantations, Kidney” OR “Transplantation, Renal” OR “Renal Transplantation” OR “Renal Transplantations” OR “Transplantations, Renal” OR “Grafting, Kidney” OR “Kidney Grafting” OR “Kidney Transplantation” OR “MeSH descriptor:Kidney Transplantation 1 tree(s) exploded”) AND (“male” OR “female” OR “gender” OR “Factor, Sex” OR “Factors, Sex” OR “Sex Factor” OR “Sex Factors” OR “MeSH descriptor:Sex Factors explode all trees”) AND “Characteristic, Sex” OR “Characteristics, Sex” OR “Sex Characteristic” OR “Sex Differences” OR “Difference, Sex” OR “Differences, Sex” OR “Sex Difference” OR “Sex Characteristics” OR “MeSH descriptor:Sex Characteristics explode all trees”) AND (“graft rejection OR MeSH descriptor:Graft Rejection explode all trees”) AND (“survival rate” “MeSH descriptor:Survival Rate explode all trees”) AND “graft survival”
LILACS	87	(tw:(transplantation kidney)) OR (tw:(transplantation renal)) AND (tw:(gender differences)) OR (tw:(gender Characteristics)) OR (tw:(Sex Characteristics)) OR (tw:(sex differences)) AND (tw:(survival rate)) AND (tw:(survival graft))

After duplicate studies were excluded, two independent reviewers selected the references in three phases: analysis of titles, abstracts, and full texts. The disagreements were settled by a third reviewer. The data – including methodological quality, subject information, treatment length, and patient and graft survival rates – were extracted and collected in duplicate in a Microsoft Excel 2010 spreadsheet.

Methodological quality evaluations were independently conducted by the reviewers, and related disagreements were settled through the consensus among reviewers. Newcastle-Ottawa scale^b^ for observational studies was used. In this scale, each study is evaluated in three dimensions: selection of study groups; the comparability among groups; and the ascertainment of either the exposure or outcome of interest. Total score was up to nine stars – above six, studies are considered to be high quality.

In order to be analyzed, the studies were grouped according to the comparison of results among:

a)Donor genders – male donors (MD) and female donors (FD);

b)Recipient genders – male recipients (MR) and female recipients (FR);

c)Donor-recipient genders – male donor-male recipient transplants (MD-MR), male donor-female recipient transplants (MD-FR), female donor-female recipient transplants (FD-FR), female donor-male recipient transplants (FD-MR).

The study data were combined using randomized effects model in Metaview module of Review Manager software, version 5.3. The results were presented as relative risk for dichotomous variables, with a confidence interval of 95%. Analyses with I^2^ > 40.0% and p-value of Chi-squared test < 0.10 were considered to be significant heterogeneity. A sensitivity analysis was conducted to investigate heterogeneity causes, with the exclusion of one study at a time, with changes being verified in the values of I^2^ and p.

The outcomes that were evaluated in the meta-analysis were graft survival and patient survival per follow-up period (one, two, three, five, eight, 10 years or more).

## RESULTS

A total of 5,993 publications were initially identified in the electronic databases, and seven through manual searches, all adding up to 6,000 publications. Of these, 500 publications were excluded because of the participant type, 5,251 due to study type and 177 due to the intervention. The main causes for excluded studies were: studies that did not analyze the outcome of interest (patient and graft survival rates), ones that did not include kidney transplants, review, pharmacokinetic, pharmacoeconomic studies, among others. After duplicate publications were eliminated and the reviewers conducted their analyses, 29 cohort studies were included, which involved 765.753 patients. Among those, six studies compared the measurements from results involving donor genders (MD and FD); eight studies, involving recipient genders (MR and FR), and for 20 others, results involving donor-recipient genders (MD-MR, MD-FR, FD-FR, FD-MR) (Figure). One study was included in donor, recipient, and donor-recipient gender comparisons;^5^ another one^14^ was included in the comparison between recipient and donor-recipient genders; and two studies, in the comparison between donor and donor-recipient genders.^3^ ^,^ ^25^

**Figure:**
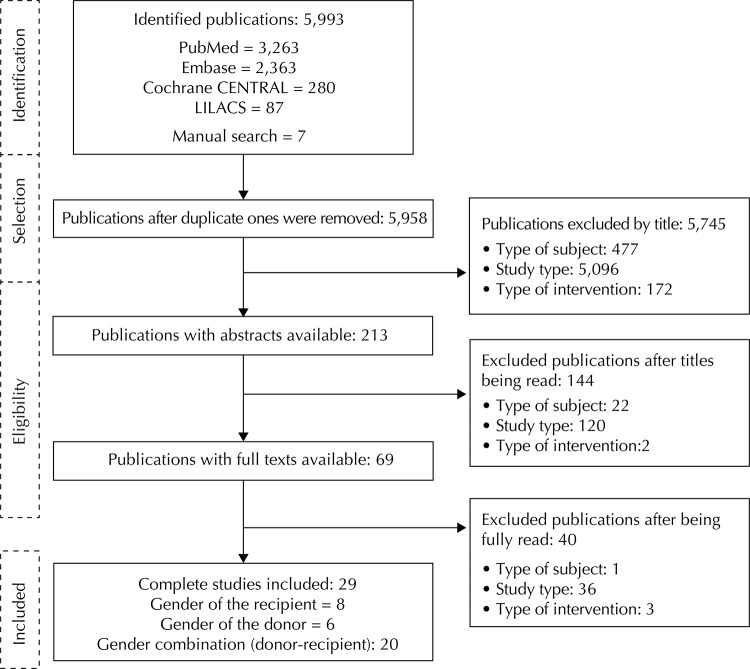
Flowchart of study selection for systematic review.

Out of the 29 observational studies included, 28 were retrospective and one was prospective.^38^ Most studies have not reported average follow-up periods, and the data from the cohorts were collected from 1978 to 2009. In the comparison between donor genders, 9,673 subjects were evaluated in the six studies. In the comparison between recipient genders, 84,070 subjects were evaluated in the eight studies included. In the comparison between donor-recipient genders, 672,010 subjects were evaluated in the 20 studies included. In regards to types of donors, 12 studies evaluated deceased donors, eight evaluated living donors, and nine evaluated both kinds (living and deceased) (Table 2).

**Table 2:** General characteristics of studies included in comparisons between genders.

Study	Comparison between genders	Number of patients	Country where the study was conducted	Type of donor	Donor age (years)	Recipient age (years)	Immunosuppressive therapy	Collection period	Average supervision type (months)	Newcastle Scale
Neugarten et al^25^ (1996)	Donor and donor-recipient	651	USA	Both	NR	NR	Ciclosporin	1979 to 1994	NR	7
Buchler et al^4^ (1997)	Donor	354	France	Deceased	NR^a^	49	Multiple	1985 to 1995	NR	6
Busson e Benoit^5^ (1997)	Recipient. donor. and donor-recipient	6,889	France	Deceased	NR^a^	NR^a^	NR	1989 to 1992	NR	7
Valdes et al^41^ (1997)	Donor	858	Spain	Deceased	NR^a^	NR^a^	NR	1981 to 1995	NR	7
Ben Hamida et al^3^ (1999)	Donor and donor-recipient	182	Tunisia	Living	39.3	28.1	Multiple	1986 to 1998	NR	7
Oien et al^29^ (2007)	Donor	739	Norway	Living	NR^a^	NR^a^	Multiple	1994 to 2004	55.1	8
Sánches Garcia et al^35^ (1989)	Recipient	760	Spain	Deceased	NR	NR	Ciclosporin	1978 to 1988	NR	7
Nyberg et al^27^ (1997)	Recipient	1,000	Sweden	Both	NR^a^	NR^a^	Ciclosporin + prednisolone	1985 to 1993	73	8
Avula et al^2^ (1998)	Recipient	431	India	Living	43.18	33.87	Multiple	NR	9	6
Meier-KriescheH et al^22^ (2001)	Recipient	73,477	USA	Both	NR^a^	NR^a^	Multiple	1988 to 1997	NR	8
Inoue et al^14^ (2002)	Recipient and donor-recipient	205	Japan	Both	NR^a^	NR^a^	Cisclosporin or FK506	1987 to 2000	NR	7
Moosa^23^ (2003)	Recipient	542	South Africa	Deceased	NR^a^	37	Multiple	1976 to 1999	75.6	7
Chen et al^6^ (2013)	Recipient	766	China	Both	NR^a^	NR^a^	Multiple	1988 to 2009	NR	7
Ellison etal^8^ (1994)	Donor-recipient	3,314	USA	Both	NR	NR	NR	1987 to 1992	NR	8
Shaheen et al^38^ (1998)^b^	Donor-recipient	406	Saudi Arabia	Living	31.3	34.3	Ciclosporin	NR	55.2	7
Vereerstraeten et al^42^ (1999)	Donor-recipient	741	Belgium	Deceased	34.8	36.9	Multiple	1983 to 1997	NR	7
Zeier et al^43^ (2002)	Donor-recipient	119,195	49 countries	Both	38.3	44.3	NR	1985 to 2000	NR	8
Kayler et al^16^ (2003)	Donor-recipient	30,258	USA	Living	NR^a^	NR^a^	NR	1990 to 1999	NR	8
Kwon e Kwak^19^ (2004)	Donor-recipient	614	South Korea	Living	NR^a^	NR^a^	NR	1979 to 2002	NR	7
Pugliese et al^34^ (2005)	Donor-recipient	3,233	Italy	Deceased	42.1	44.5	NR	1995 to 2000	NR	8
Jacobs et al^15^ (2007)	Donor-recipient	730	USA	Living	39.7	46.4	NR	1979 to 1994	NR	8
Gratwohl et al^12^ (2008)	Donor-recipient	195,516	45 countries	Deceased	38	44.7	NR	1985 to 2004	67.2	8
Kim e Gill^17^ (2009)	Donor-recipient	117,877	USA	Deceased	NR^a^	NR^a^	NR	1990 to 2004	NR^a^	7
Lankarani et al^20^ (2009)	Donor-recipient	2,649	Iran	Living	NR^a^	NR^a^	NR	1992 to 2005	NR	8
Shaheen et al^39^ (2010)	Donor-recipient	524	Saudi Arabia	Deceased	33.6	33.9	Múltipla	2003 to 2007	20.9	8
Głyda et al^11^ (2011)	Donor-recipient	154	Poland	Deceased	NR^a^	NR^a^	Múltipla	NR	NR	7
Zukowski et al^45^ (2011)	Donor-recipient	230	Poland	Deceased	33.1	37.6	NR	NR	NR	8
Abou-Jaoude et al^1^ (2012)	Donor-recipient	135	Lebanon	Both	NR^a^	NR^a^	Múltipla	1998 to 2007	NR	7
Tan et al^40^ (2012)	Donor-recipient	188,507	USA	Both	NR^a^	NR^a^	NR	1988 to 2006	NR	8

NR: not reported; NR^a^: not reported in the expected way

^b^ Prospective cohort.

The majority of subjects (donor, recipient, and donor-recipient) were males, for all gender comparisons. All studies included evaluated graft survival rates, but only eight of them evaluated patient survival rates.^1^ ^,^ ^14^ ^,^ ^20^ ^,^ ^22^ ^,^ ^23^ ^,^ ^27^ ^,^ ^39^ ^,^ ^43^

Out of the 29 studies included, two of them were observed to have scores of six as per New-Castle Ottawa scale;^2^ ^,^ ^4^ 14 (48,3%) of them had score seven; and 13, eight (Table 2).

Out of the six studies included in the systematic review for donor gender comparison, five of them were included in the meta-analysis for graft survival outcome.^3^ ^-^ ^5^ ^,^ ^29^ ^,^ ^41^ The study by Neugarten et al^25^ was not found to have enough numerical data for the quantitative analysis. The studies included in the donor gender comparison did not evaluate patient survival.

Regarding graft survival, the relative risks (RR), as grouped chronologically according to follow-up periods of one, two, three, five, and ten years were, respectively, 1.02 (95%CI 0.97;1.07; p = 0.43; I^2^ = 70.0%), 1.03 (95%CI 1.00;1.07; p = 0.07; I^2^ = 27.0%), 1.02 (95%CI 0.94;1.12; p = 58; I^2^ = 67.0%), 0.89 (95%CI 0.79;1.00; p = 0.06; I^2^ = 0%), 0.86 (95%CI 0.73;1.02; p = 0.09; I^2^ = 11.0%;), and 0.82 (95%CI 0.68;0.98; p = 0.03; I^2^ = 0%). Only at the 10-year follow-up period was the difference significant for graft survival, favoring male donors. Heterogeneity was high and significant for follow-up periods of one and three years (Table 3).

**Table 3:** Summary of meta-analyses for survival of grafts according to donor genders (FD or MD) and recipient genders (FR or MR); and survival of patients according to kidney transplant recipient genders.

Outcome/Time	Studies	Subjects	RR	95%CI	p^a^	I^2^ (%)^b^
Graft survival	FD *versus* MD					
1 year	^3,5,30^	7,478	1.02	0.97;1.07	0.43	70.0^c^
2 years	^5,30,41^	8,154	1.03	1.00;1.07	0.07	27.0
3 years	^5,30,41^	8,154	1.02	0.94;1.12	0.58	67.0^c^
5 years	^3,30^	589	0.89	0.79;1.00	0.06	0
8 years	^4,30^	761	0.86	0.73;1.02	0.09	11.0
10 years	^3,30^	589	0.82	0.68;0.98	0.03	0
Graft survival	FR *versus* MR					
1 year	^2,5,10^	8,348	1.01	0.99;1.03	0.30	0
5 years	^2,6,15^	1,402	0.99	0.88;1.12	0.88	75.0^c^
10 years and older	^15,24^	747	1.23	0.68;2.24	0.50	95.0^c^
Patient survival	FR *versus* MR					
10 years and older	^15,24^	747	0.96	0.67;1.37	0.81	95.0^c^

RR: relative risk

^a^ Value of p < 0.10 of Z-test for all effects.

^b^ Value of I2 > 40.0% indicates statistical heterogeneity among studies.

^c^ Significant heterogeneity (p < 0.10).

Out of the eight studies included in the systematic review for recipient gender comparison, six were included in the meta-analysis for graft survival outcome.^2^ ^,^ ^5^ ^,^ ^6^ ^,^ ^14^ ^,^ ^23^ ^,^ ^35^ The studies by Nyberg et al^27^ and Meier-Kriesche et al^22^ were not found to have numerical data in order to be included in the quantitative analysis. Thus, meta-analyses were conducted for monitored periods of one, five, and 10 years or more.

In the meta-analysis regarding graft survival for one year, four comparisons of three studies were included. In the study by Sánchez Garcia et al,^35^ (1989) the influence from immunosuppressive therapy was compared to genders in two groups: Sánchez Garcia et al^35^ (1989a), ciclosporin-treated; and Sánchez Garcia et al^35^ (1989b), ciclosporin-untreated.

Regarding graft survival, the relative risks (RR), as grouped chronologically according to follow-up periods of one, five, and 10 years or more were, respectively, 1.01 (95%CI 0.99;1.03; p = 0.30; I^2^ = 0%), 0.99 (95%CI 0.88;1.12; p = 0.88; I^2^ = 75.0%), 1.23 (95%CI 0.68;2.24; p = 0.50; I^2^ = 95.0%). No significant differences were found for any of the follow-up periods, and no recipient genders were highlighted among the groups. Heterogeneity was high and significant for follow-up periods of five and 10 years or more (Table 3).

Regarding patient survival, two studies were included in the related meta-analysis.^14^ ^,^ ^24^ The meta-analysis was conducted for the follow-up period of 10 years or more, heterogeneity was high and the difference was not significant (RR = 0.96; 95%CI 0.67;1.37; p = 0.81; I^2^ = 95.0%) (Table 3).

In order to evaluate donor-recipient genders, six comparisons were analyzed: MD-MR *versus* FD-MR, MD-MR *versus* FD-FR, MD-MR *versus* MD-FR, MD-FR *versus* FD-FR, MD-FR *versus* FD-MR, FD-FR *versus* FD-MR. Regarding graft survival outcome, 13 studies were included^1^ ^,^ ^5^ ^,^ ^8^ ^,^ ^11^ ^,^ ^15^ ^-^ ^17^ ^,^ ^19^ ^,^ ^20^ ^,^ ^38^ ^-^ ^40^ ^,^ ^43^ in the meta-analyses (Table 4).

**Table 4:** Summary of meta-analyses for survival of grafts according to kidney transplant donor-recipient genders.

General characteristics		MD-MR *versus* MD-FR					MD-FR *versus* FD-FR				
Time	Studies	Number of patients	RR	95%CI	p^a^	I^2^ (%)^b^	Number of patients	RR	95%CI	p^a^	I^2^ (%)^b^
1 year	^1,5,8,15,16,17,20,39,40^	211,025	1.03	1.01;1.05	< 0.01	92.0^c^	181,223	1.02	1.01. 1.04	< 0.01	83.0^c^
2 years	^5,8,15,20^	8,411	1.05	1.03;1.08	< 0.01	25.0	7,575	1.02	1.00;1.04	0.10	9.0
3 years	^5,8,15,17,20,39^	79,979	1.06	1.02;1.09	< 0.01	72.0^c^	69,993	1.05	1.01;1.09	0.02	73.0^c^
5 years	^11,16,17,19,20,38,40^	204,668	1.15	1.00;1.33	0.05	100^c^	175,602	1.02	1.01;1.03	< 0.01	50.0^c^
10 year	^17,40,43^	258,63	1.08	1.05;1.11	< 0.01	91.0^c^	223,404	1.02	0.97;1.07	0.43	97.0^c^

General characteristics		MD-MR *versus* MD-FR					MD-FR *versus* FD-FR				

Time	Studies	Number of patients	RR	95%CI^a^	p^b^	I^2^ (%)^c^	Number of patients	RR	95%CI^a^	p^b^	I^2^ (%)^c^

1 year	^1,5,8,15,16,17,20,39,40^	196,191	1.01	1.01;1.02	< 0.01	67.0^c^	139,859	1.01	0.99;1.02	0.30	77.0^c^
2 years	^5,8,15,20^	9,086	1.01	0.99;1.03	0.32	0	5,171	1.01	0.98;1.03	0.61	0
3 years	^5,8,15,17,20,39^	80,495	1.03	1.00;1.06	0.07	76.0^c^	51,994	1.01	1.00;1.02	< 0.01	0
5 years	^11,16,17,19,20,38,40^	189,508	1.00	0.97;1.03	0.98	91.0^c^	135,798	1.01	1.00;1.03	0.11	66.0^c^
10 year	^17,40,43^	246,909	0.96	0.86;1.06	0.38	99.0^c^	166,817	1.04	1.02;1.06	< 0.01	80.0^c^

General characteristics		MD-FR *versus* FD-MR					FD-FR *versus* FD-MR				

Time	Studies	Number of patients	RR	95%CI^a^	p^b^	I^2^ (%)^c^	Number of patients	RR	95%CI^a^	p^b^	I^2^ (%)^c^

1 year	^1,5,8,15,16,17,20,39,40^	169,666	1.01	1.00;1.03	0.15	90.0^c^	154,693	1.01	0.99;1.02	0.45	80.0^c^
2 years	^5,8,15,20^	5,679	1.05	1.01;1.10	0.01	36.0	4,496	1.03	0.99;1.07	0.18	51.0^c^
3 years	^5,8,15,17,20,39^	61,980	1.03	1.00;1.05	0.04	44.0^c^	51,478	1.01	0.98;1.04	0.53	44.0
5 years	^11,16,17,19,20,38,40^	164,864	1.18	1.03;1.35	0.02	100^c^	150,958	1.14	0.99;1.31	0.07	100^c^
10 year	^17,40,43^	202,176	1.10	1.01;1.21	0.03	99.0^c^	176,671	1.06	0.98;1.14	0.14	98.0^c^

RR: relative risk

^a^ Value of p < 0.10 of Z-test for all effects.

^b^ Value of I2 > 40.0% indicates statistical heterogeneity among studies.

^c^ Significant heterogeneity (p < 0.10).

The remaining studies were not found to have enough data for the quantitative analysis.^3^ ^,^ ^10^ ^,^ ^12^ ^,^ ^14^ ^,^ ^25^ ^,^ ^42^ ^,^ ^45^ For the patient survival outcome, two studies were included in the meta-analysis.^1^ ^,^ ^20^

The studies by Ellison et al^8^ and Tan et al^40^ separately evaluated transplants from living and deceased donors, and the total numbers of events and subjects in each study were included in the meta-analysis, considering living and deceased donors. In the study by Abou-Jaoude et al,^1^ rates regarding general graft survival and graft survival as interrupted by death with functioning graft were calculated. The uninterrupted graft survival rate was the one used in the meta-analysis.

In the MD-MR *versus* FD-MR comparison, the RRs as grouped for graft survival in a chronological order of follow-up periods of one, two, three, five, and 10 years were, respectively, 1,03 (95%CI 1.01;1.05; p = 0.0002; I^2^ = 92.0%), 1.05 (95%CI 1.03;1.08; p < 0.0001; I^2^ = 25.0%), 1.06 (95%CI 1.02;1.09; p = 0.0008; I^2^ = 72.0%), 1.15 (95%CI 1.00;1.33; p = 0.05; I^2^ = 100%), and 1.08 (95%CI 1.05;1.11; p < 0.00001; I^2^ = 91.0%). The graft survival rate was significantly higher in all follow-up periods evaluated, and it favored MD-MR pair. Heterogeneity was high and significant for all periods, except for the two-year follow-up period. The patient survival analysis was only conducted for the one-year follow-up period, and no pairs were observed to be favored (RR = 0.99; 95%CI 0.96;1.02; p = 0.52; I^2^ = 0.0%).

In the MD-MR *versus* FD-FR comparison, the RRs as grouped for graft survival in a chronological order of follow-up periods of one, two, three, five, and 10 years were, respectively, 1.02 (95%CI 1.01;1.04; p = 0.0008; I^2^ = 83.0%), 1.02 (95%CI 1.00;1.04; p < 0.10; I^2^ = 9.0%), 1.05 (95%CI 1.01;1.09; p = 0.02; I^2^ = 73.0%), 1.02 (95%CI 1.01;1.03; p = 0.0004; I^2^ = 50.0%), and 1.02 (95%CI 0.97;1.07; p = 0.43; I^2^ = 97.0%). The graft survival rate was significantly higher in follow-up periods of one, three, and five years, and it favored MD-MR pair. Heterogeneity only was not significant for the two-year follow-up period. The patient survival meta-analysis was observed to favor none of the pairs (RR = 0.98; 95%CI 0.95;1.01; p = 0.21; I^2^ = 0%).

In the MD-MR *versus* MD-FR comparison, the RRs as grouped for graft survival in a chronological order of follow-up periods of one, two, three, five, and 10 years were, respectively, 1.01 (95%CI 1.01;1.02; p = 0.0009; I^2^ = 67.0%), 1.01 (95%CI 0.99;1.03; p = 0.32; I^2^ = 0%), 1.03 (95%CI 1.00;1.06; p = 0.07; I^2^ = 76.0%), 1.00 (95%CI 0.97;1.03; p = 0.98; I^2^ = 91.0%), and 0.96 (95%CI 0.86;1.06; p = 0.38; I^2^ = 99.0%). The graft survival rate was only significantly higher for the one-year follow-up period, favoring the MD-MR pair. Heterogeneity only was not significant for the two-year follow-up period. The patient survival meta-analysis was observed to favor none of the pairs (RR = 1.00; 95%CI 0.98;1.03; p = 0.86; I^2^ = 0%).

In the MD-FR *versus* FD-FR comparison, the RRs as grouped for graft survival in a chronological order of follow-up periods of one, two, three, five, and 10 years were, respectively, 1.01 (95%CI 0.99;1.02; p = 0.30; I^2^ = 77.0%), 1.01 (95%CI 0.98;1.03; p = 0.61; I^2^ = 0%), 1.01 (95%CI 1.00;1.02; p = 0.0007; I^2^ = 0%), 1.01 (95%CI 1.00;1.03; p = 0.11; I^2^ = 66.0%), and 1.04 (95%CI 1.02;1.06; p = 0.0003; I^2^ = 80.0%). The graft survival rate was significantly higher in follow-up periods of three and ten years, and it favored MD-FR pair. Heterogeneity only was not significant for the two and three-year follow-up periods. The patient survival meta-analysis was observed to favor none of the pairs (RR = 0.98; 95%CI 0.94;1.01; p = 0.19; I^2^ = 0%).

In the MD-FR *versus* FD-FR comparison, the RRs as grouped for graft survival in a chronological order of follow-up periods of one, two, three, five, and 10 years were, respectively, 1.01 (95%CI 1.00;1.03; p = 0.15; I^2^ = 90.0%), 1.05 (95%CI 1.01;1.10; p = 0.01; I^2^ = 36.0%), 1.03 (95%CI 1.00;1.05; p = 0.04; I^2^ = 44.0%), 1.18 (95%CI 1.03;1.35; p = 0.02; I^2^ = 100%), and 1.10 (95%CI 1.01;1.21; p = 0.03; I^2^ = 99.0%). The graft survival rate was significantly higher for all follow-up periods, except for the one-year one, favoring MD-FR pair. Heterogeneity was high and significant for all periods, except for the two-year follow-up period. The patient survival meta-analysis was observed to favor none of the pairs (RR = 0.99; 95%CI 0.95;1.02; p = 0.44; I^2^ = 0%).

In the FD-FR *versus* FD-MR comparison, the RRs as grouped for graft survival in a chronological order of follow-up periods of one, two, three, five, and 10 years were, respectively, 1.01 (95%CI 0.99;1.02; p = 0.45; I^2^ = 80.0%), 1.03 (95%CI 0.99;1.07; p = 0.18; I^2^ = 51.0%), 1.01 (95%CI 0.98;1.04; p = 0.53; I^2^ = 44.0%), 1.14 (95%CI 0.99;1.31; p = 0.07; I^2^ = 100%), and 1.06 (95%CI 0.98;1.14; p = 0.14; I^2^ = 98.0%). There were no significant differences regarding graft survival considering all follow-up periods. Heterogeneity was high and significant for all periods, except for the three-year follow-up period. The patient survival meta-analysis was observed to favor none of the pairs (RR = 1.01; 95%CI 0.97;1.06; p = 0.54; I^2^ = 0%).

## DISCUSSION

This meta-analysis evaluated the influences of donor genders, recipient genders, and the donor-recipient combination in regards to kidney transplant patient and graft survival rates.

In the comparison between donor genders, 9,022 subjects were evaluated in the meta-analysis. Donor genders were not found to favor gender rates in the evaluation of one, two, three, five, and eight-year follow-up periods (p < 0.05). Ten-year follow-up period was the only observed to differ significantly, favoring male donors (p = 0.03). The study by Muller^24^ concluded that kidney grafts from male patients work better than the ones from female donors in the long run. Several studies suggest that, in those cases, the grafts from female donors are more antigenic, which may explain the lower survival rates.^22^ ^,^ ^30^ ^,^ ^31^ ^,^ ^36^

In the comparison between recipient genders in all follow-up periods, 9,593 subjects were evaluated in the meta-analysis, and no significant differences were observed regarding graft survival. Patient survival analysis considered 747 patients. No significant differences were found among the studies. The study by Busson and Benoit^5^ evaluated the influence from recipient and donor genders and the donor-recipient combination. Recipient genders were the only ones for which significant differences were not found.

The comparison between donor-recipient genders included 471,252 patients. MD-MR pair should be highlighted for having been observed to have the best results in all comparisons (MD-MR *versus* FD-MR, MD-MR *versus* FD-FR, MD-MR *versus* MD-FR); that is, kidney transplants from male donors to male recipients were found to have the best graft survival rates. Nonetheless, FD-MR pair was found to have the worst results (FD-MR *versus* MD-MR, FD-MR *versus* MD-FR), except for the FD-MR *versus* FD-FR comparison. Those results are comparable with other reviews.^7^ ^,^ ^44^

The assessment of differences between genders is important to improve transplant results. Men and women have different biological factors, different body conditions, hormone circumstances, and immune responses, as well as different metabolic and functional demands, which can influence kidney transplant results.^44^ Gender incompatibilities in FD-MR pair are argued to negatively influence graft survival due to kidney sizes and their numbers of nephrons. The ratio between graft and recipient weights is an important one.^26^ ^,^ ^33^

Giral et al^10^ analyzed the consequences from kidney mass reduction following kidney transplants, and they concluded kidney graft mass to impact glomerular filtration and proteinuria rates. The authors suggest that great kidney-to-recipient weight ratios be avoided, once that might significantly influence long-term kidney function.

That meta-analysis only included cohort studies. One of the limitations from systematic reviews with meta-analyses of observational studies regards to the selection bias that is intrinsic to this study design and to uncontrolled confounding factors. Observational cohort studies are the ones conducted in the real world, under conditions uncontrolled for. In regards to that, differences were observed in the subject numbers among the groups, types of donors (living, deceased, or both), numbers of transplants, monitored periods, among others. Despite that, observational studies are observed to have the advantages of gathering a large number of patients and best representing the real world.

Another limitation in the interpretation of results was the statistical heterogeneity among studies, which was found in the meta-analyses. The small number of studies included in the comparisons, and the lack of complete, accurate information in the studies made it difficult to account for heterogeneity sources. Most studies were not observed to include immunosuppressive therapies, ages (donors and recipients), or monitored periods. The sensitivity analysis, in which studies were included and excluded for each comparison, in general, has not altered the directions of outcomes, having changes of small relevance in heterogeneity values. It has not provided information on the possible causes for heterogeneity either.

However, the results from this review can be considered for decision-making by medical teams responsible for transplants in the clinical practice, once it represents the best level of evidence regarding the topic.

Gender incompatibilities must be avoided whenever possible. Genders must be considered as criteria in the choices regarding allocation of organs from donors and to recipients. Gender combinations may make a difference in survival rates. Nonetheless, that reality is utopic in the clinical practice, due to the scarcity of donors and the increase in the number of patients on waiting lists for transplants. A change in that scenario is required in order to improve the allocation of organs.

In conclusion, recipient and donor genders, when evaluated isolatedly, do not influence patient or graft survival rates. However, combinations between donor-recipient genders may be a determining factor for graft survival, favoring MD-MR pair.

## Footnotes

a Higgins JPT, Green S, editors. Cochrane handbook for systematic reviews of interventions: version 5.1.0. London: The Cochrane Collaboration; 2011 [cited 2014 Feb 6]. Available from: http://www.cochrane-handbook.org

b Wells GA, Shea B, O’Connelll D, Peterson J, Welch V, Losos M, et al. The Newcastle-Ottawa Scale (NOS) for assessing the quality of nonrandomised studies in meta-analyses. Ottawa: Ottawa Hospital Research Institute; 2014 [cited 2014 Feb 6]. Available from: http://www.ohri.ca/programs/clinical_epidemiology/oxford.asp
