# Supplementary material for: Gender in the allocation of organs in kidney transplants: meta-analysis
Source: Rev Saude Publica. 2015 Sep 23;49:68. doi: 10.1590/S0034-8910.2015049005822 (PMC4587823; doi:10.1590/S0034-8910.2015049005822)
Supplement: Translation

# Translation: Gênero na alocação de órgãos no transplante renal: metanálise

**Authors:** Erika Vieira Almeida e Santiago, Micheline Rosa Silveira, Vânia Eloisa de Araújo, Katia de Paula Farah, Francisco de Assis Acurcio, Maria das Graças Braga Ceccato

**Affiliations:** I Programa de Pós-Graduação em Medicamentos e Assistência Farmacêutica. Faculdade de Farmácia. Universidade Federal de Minas Gerais. Belo Horizonte, MG, Brasil; II Departamento de Odontologia. Instituto de Ciências Biológicas e da Saúde. Pontifícia Universidade Católica de Minas Gerais. Belo Horizonte, MG, Brasil; III Departamento de Clínica Médica. Faculdade de Medicina. Universidade Federal de Minas Gerais. Belo Horizonte, MG, Brasil

**Keywords:** Transplante de Rim, Distribuição por Sexo, Gênero e Saúde, Prognóstico, Metanálise

## Abstract

**OBJETIVO:**

Analisar se o gênero influencia os resultados da sobrevida do enxerto e do paciente no transplante renal.

**MÉTODOS:**

Revisão sistemática com metanálise de estudos de coorte disponíveis nas bases de dados Medline (PubMed), Lilacs, Central e Embase, incluindo busca manual e na literatura cinzenta. A seleção dos estudos e a coleta dos dados foram realizadas em duplicata por revisores independentes e as discordâncias foram resolvidas por um terceiro revisor. A sobrevida do enxerto e do paciente foram avaliadas como medidas de efetividade. A metanálise foi conduzida no *softwareReview Manager* ^®^ 5.2, aplicando o modelo de efeitos aleatórios. Foram avaliadas as comparações entre gênero de receptor, doador e doador-receptor.

**RESULTADOS:**

Foram incluídos 29 estudos envolvendo 765.753 pacientes. Em relação à sobrevida do enxerto, aqueles provenientes de doadores masculinos apresentaram maior sobrevida quando comparado com aqueles de doadores femininos, somente no tempo de acompanhamento de 10 anos. A comparação entre gênero de receptor não apresentou diferença significativa em nenhum tempo de acompanhamento avaliado. Na avaliação entre gênero de doador-receptor houve favorecimento estatisticamente significante para doador masculino-receptor masculino. Não houve diferença, estatisticamente significante, na sobrevida do paciente para as comparações entre gênero em todos os períodos de acompanhamento avaliados.

**CONCLUSÕES:**

A análise quantitativa dos estudos sugere que o gênero do doador ou do receptor, avaliado isoladamente, não influencia na sobrevida do enxerto e paciente. Contudo, a combinação entre gênero de doador-receptor pode ser fator determinante para a sobrevida do enxerto.

## INTRODUÇÃO

O transplante renal é considerado a melhor alternativa terapêutica para os portadores de doença renal crônica avançada.^13^ ^,^ ^18^ ^,^ ^32^ Diferenças entre gênero em relação ao transplante renal são relatadas na literatura, observadas na prática clínica nas últimas décadas, e afeta os resultados do transplante, como nas rejeições agudas e crônicas e nas sobrevidas do enxerto e dos pacientes. As mulheres têm menor acesso ao transplante, apresentam risco aumentado para rejeição aguda e diminuído para rejeição crônica, sendo esses riscos acentuados pelo envelhecimento.^9^ ^,^ ^22^ Em contrapartida, as mulheres representam cerca de 65,0% dos doadores vivos de rins.^7^ ^,^ ^37^ A etiologia dessas diferenças ainda é desconhecida, mas, provavelmente, reflete diferenças hormonais, imunológicas, envelhecimento e preconceitos.^7^ ^,^ ^22^ ^,^ ^28^

A sobrevida após o transplante renal é maior entre as mulheres,^9^ ^,^ ^11^ mas os dados são discordantes na literatura. Em estudo na África do Sul, observou-se pior sobrevida entre as mulheres, mas não houve diferenças significativas entre gênero na sobrevida do enxerto.^23^ Em outro estudo, não foram observadas diferenças entre gênero na sobrevida do paciente e do enxerto.^27^

Conhecer as diferenças entre gênero é necessário para identificar possíveis barreiras para obtenção dos resultados ideais e desenvolvimento de intervenções que as superem. Esta revisão, ao focalizar essas diferenças entre gênero na efetividade clínica da terapia imunossupressora de manutenção do transplante renal, poderá promover melhor compreensão, proporcionar cuidados à saúde mais eficientes, contribuir para a elaboração de protocolos clínicos e promover melhores resultados em longo prazo para os pacientes.

O objetivo desta revisão foi analisar se gênero influencia a sobrevida de enxerto e paciente no transplante renal.

## MÉTODOS

Esta revisão foi realizada de acordo com as recomendações da *Cochrane Collaboration Handbookv*.^a^ O artigo foi preparado segundo o *Preferred Reporting Items for Systematic reviews and Meta-Analysis* (PRISMA).^21^

Foram selecionados estudos observacionais, do tipo coorte, realizados em pacientes que receberam transplantes renais de doadores vivos ou cadáver, pela primeira vez ou por mais vezes, que abordaram as diferenças entre gênero relacionadas às características pré-transplantes e que apresentaram resultados de sobrevida do enxerto, do paciente ou de ambos. Os critérios de exclusão foram estudos não relacionados aos imunossupressores de manutenção do transplante renal, estudos farmacocinéticos, de avaliação econômica, de revisão e estudos realizados em animais.

Foi realizada busca eletrônica de artigos publicados até dezembro de 2013 nas bases de dados Medline (PubMed), Literatura Latino-Americana e do Caribe em Ciências da Saúde (Lilacs), *Cochrane Controlled Trials Databases* (Central), Embase. Também foi feita busca manual nas listas de referências de todos os estudos selecionados da revisão sistemática publicada.^44^ Foi também realizada busca por estudos da literatura cinzenta: no banco de teses e dissertações da Coordenação de Aperfeiçoamento de Pessoal de nível superior (Capes), da Biblioteca Digital Brasileira de Teses e Dissertações e da Biblioteca Digital de Teses e Dissertações da Universidade de São Paulo. Não houve restrições quanto à data e ao idioma das publicações. A Tabela 1 descreve a estratégia de busca utilizada em cada base de dados consultada.

**Tabela 1:** Estratégia de busca bibliográfica para estudos observacionais realizada em cada base eletrônica, em 12/12/2013.

Base eletrônica	Estudos	Estratégia de busca
Medline (via PubMed)	3.263	((((((((((((((Transplantation, Kidney) OR Kidney Transplantations) OR Transplantations, Kidney) OR Transplantation, Renal) OR Renal Transplantation) OR Renal Transplantations) OR Transplantations, Renal) OR Grafting, Kidney) OR Kidney Grafting)) OR (Kidney Transplantation) OR (“Kidney Transplantation”[Mesh) AND (((((male[Title/Abstract) OR female[Title/Abstract) OR gender[Title/Abstract)) OR (((((((((Factor, Sex) OR Factors, Sex) OR Sex Factor)) OR (Sex Factors)) OR (“Sex Factors”[Mesh)) OR (((((((Characteristic, Sex) OR Characteristics, Sex) OR Sex Characteristic) OR Sex Differences) OR Difference, Sex) OR Differences, Sex) OR Sex Difference)) OR (Sex Characteristics)) OR (“Sex Characteristics”[Mesh))) AND Humans[Mesh)) AND ((((“Cohort Studies”[Mesh) OR (cohort study) OR (studies, cohort) OR (study, cohort) OR (concurrent studies) OR (studies, concurrent) OR (concurrent study) OR (study, concurrent) OR (historical cohort studies) OR (studies, historical cohort) OR (cohort studies, historical) OR (cohort study, historical) OR (historical cohort study) OR (study, historical cohort) OR (analysis, cohort) OR (analysis, cohort) OR (cohort analyses) OR (cohort analysis) OR (closed cohort studies) OR (cohort studies, closed) OR (closed cohort study) OR (cohort study, closed) OR (study, closed cohort) OR (studies, closed cohort) OR (incidence studies) OR (incidence study) OR (studies, incidence) OR (study, incidence) OR (cohort studies) OR (cohort) OR (cohort analysis) OR (cohort study) OR (prospective cohort) OR (retrospective cohort) OR (retrospective cohort study) OR (prospective cohort study) OR (“Follow-Up Studies”[Mesh) OR (follow up studies) OR (follow-up study) OR (studies, follow-up) OR (study, follow-up) OR followup studies OR (followup study) OR (studies, followup) OR (study, followup) OR (“Epidemiologic Studies”[Mesh OR “Cross-Sectional Studies”[Mesh OR “Retrospective Studies”[Mesh OR “Longitudinal Studies”[Mesh OR “Prospective Studies”[Mesh))) OR ((case* AND and control*[Text Word))))
Embase	2.363	‘kidney transplantation’/exp ANDembase/lim AND ‘gender and sex’/exp ANDembase/lim OR ‘sex difference’/exp ANDembase/lim OR ‘gender’/exp ANDembase/lim OR ‘sex ratio’/exp ANDembase/lim AND (‘cohort analysis’/de OR ‘comparative study’/de OR ‘control group’/de OR ‘controlled study’/de OR ‘human’/de OR ‘observational study’/de OR ‘outcomes research’/de OR ‘prospective study’/de OR ‘retrospective study’/de)
Central	280	(“Transplantation, Kidney” OR “Kidney Transplantations” OR “Transplantations, Kidney” OR “Transplantation, Renal” OR “Renal Transplantation” OR “Renal Transplantations” OR “Transplantations, Renal” OR “Grafting, Kidney” OR “Kidney Grafting” OR “Kidney Transplantation” OR “MeSH descriptor:Kidney Transplantation 1 tree(s) exploded”) AND (“male” OR “female” OR “gender” OR “Factor, Sex” OR “Factors, Sex” OR “Sex Factor” OR “Sex Factors” OR “MeSH descriptor:Sex Factors explode all trees”) AND “Characteristic, Sex” OR “Characteristics, Sex” OR “Sex Characteristic” OR “Sex Differences” OR “Difference, Sex” OR “Differences, Sex” OR “Sex Difference” OR “Sex Characteristics” OR “MeSH descriptor:Sex Characteristics explode all trees”) AND (“graft rejection OR MeSH descriptor:Graft Rejection explode all trees”) AND (“survival rate” “MeSH descriptor:Survival Rate explode all trees”) AND “graft survival”
Lilacs	87	(tw:(transplantation kidney)) OR (tw:(transplantation renal)) AND (tw:(gender differences)) OR (tw:(gender Characteristics)) OR (tw:(Sex Characteristics)) OR (tw:(sex differences)) AND (tw:(survival rate)) AND (tw:(survival graft))

Excluídas todas as duplicações, dois revisores independentes selecionaram as referências, em três fases: análise de títulos, dos resumos e dos textos completos. As discordâncias foram resolvidas por um terceiro revisor. Os dados, incluindo qualidade metodológica, informações do participante, período de duração do tratamento e sobrevida do enxerto e do paciente, foram extraídos e coletados em duplicata em planilha do Microsoft Excel 2010.

As avaliações da qualidade metodológica foram realizadas de forma independente por dois revisores e as divergências, por consenso. Foi utilizada a escala de Newcastle-Ottawa^b^ para estudos observacionais. Nesta escala, cada estudo é avaliado em três dimensões: seleção dos grupos de estudo, comparabilidade dos grupos e apuração da exposição ou resultado de interesse. A pontuação total foi de até nove estrelas, sendo que acima de seis, o estudo é considerado de alta qualidade.

Para serem analisados, os estudos foram agrupados de acordo com a comparação de resultados entre:

a) Gênero de doador – doador masculino (DM) e doador feminino (DF);

b) Gênero de receptor – receptor masculino (RM) e receptor feminino (RF);

c) Gênero de doador-receptor – doador masculino-receptor masculino (DM-RM), doador masculino-receptor feminino (DM-RF), doador feminino-receptor feminino (DF-RF), doador feminino-receptor masculino (DF-RM).

Os dados dos estudos foram combinados usando o modelo de efeitos randômicos no módulo *Metaview* do *softwareReview Manager* versão 5.3. Os resultados foram apresentados por risco relativo para as variáveis dicotômicas com intervalo de confiança de 95%. Análises com I^2 ^> 40,0% e p-valor do teste do Qui-quadrado < 0,10 foram consideradas como heterogeneidade significante. Análise de sensibilidade foi conduzida para investigar as causas da heterogeneidade, excluindo um estudo de cada vez e verificadas as mudanças nos valores de I^2^ e p.

Os desfechos avaliados na metanálise foram sobrevida do enxerto e sobrevida do paciente por tempo de seguimento (um; dois; três; cinco; oito; 10 ou mais anos).

## RESULTADOS

Inicialmente foram identificadas 5.993 publicações nas bases eletrônicas e sete pela busca manual, totalizando 6.000 publicações. Foram excluídas 500 devido ao tipo do participante, 5.251 pelo tipo de estudo e 177 devido à intervenção. As principais causas das exclusões foram: estudos que não analisavam desfecho de interesse (sobrevida do enxerto e do paciente), que não contemplavam transplante renal, estudos de revisão, farmacocinéticos e farmacoeconômico, entre outros. Após a eliminação de duplicatas e análise pelos revisores, 29 estudos de coortes foram incluídos, envolvendo 765.753 pacientes. Desses, seis estudos compararam as medidas de resultados entre gênero de doador (DM e DF); oito estudos entre gênero de receptor (RM e RF); e 20 entre combinações de gênero de doador-receptor (DM-RM, DM-RF, DF-RF, DF-RM) (Figura). Foram incluídos um estudo nas comparações entre gênero de doador, receptor e doador-receptor,^5^ um estudo^14^ na comparação entre gênero de receptor e doador-receptor e dois estudos na comparação entre gênero de doador e doador-receptor.^3^ ^,^ ^25^

**Figura:** Fluxograma da seleção de estudos para a revisão sistemática.

Dos 29 estudos observacionais incluídos, 28 eram retrospectivos e um prospectivo.^38^ A maioria dos estudos não relatou tempo médio de acompanhamento e o período de coleta de dados das coortes foi de 1978 a 2009. Na comparação entre gênero de doador, foram avaliados 9.673 participantes nos seis estudos. Na comparação entre gênero de receptor foram avaliados 84.070 participantes nos oito estudos incluídos e, na comparação entre gênero doador-receptor foram avaliados 672.010 participantes nos 20 estudos incluídos. Em relação ao tipo de doador, 12 estudos avaliaram doador cadáver, oito avaliaram doador vivo e nove, ambos (vivo e cadáver) (Tabela 2).

**Tabela 2:** Características gerais dos estudos incluídos nas comparações entre gênero.

Estudo	Comparação entre gênero	N^o^ de pacientes	País de realização do estudo	Tipo de doador	Idade do doador (anos)	Idade do receptor (anos)	Terapia imunossupressora	Período de Coleta	Tempo médio de acompanhamento (meses)	Escala Newcastle
Neugarten et al^25^ (1996)	Doador e doador-receptor	651	EUA	Ambos	NR	NR	Ciclosporina	1979 a 1994	NR	7
Buchler et al^4^ (1997)	Doador	354	França	Cadáver	NR^a^	49	Múltipla	1985 a 1995	NR	6
Busson e Benoit^5^ (1997)	Receptor, doador e doador-receptor	6.889	França	Cadáver	NR^a^	NR^a^	NR	1989 a 1992	NR	7
Valdes et al^41^ (1997)	Doador	858	Espanha	Cadáver	NR^a^	NR^a^	NR	1981 a 1995	NR	7
Ben Hamida et al^3^ (1999)	Doador e doador-receptor	182	Tunísia	Vivo	39,3	28,1	Múltipla	1986 a 1998	NR	7
Oien et al^29^ (2007)	Doador	739	Noruega	Vivo	NR^a^	NR^a^	Múltipla	1994 a 2004	55,1	8
Sánches Garcia et al^35^ (1989)	Receptor	760	Espanha	Cadáver	NR	NR	Ciclosporina	1978 a 1988	NR	7
Nyberg et al^27^ (1997)	Receptor	1.000	Suécia	Ambos	NR^a^	NR^a^	Ciclosporina + prednisolona	1985 a 1993	73	8
Avula et al^2^ (1998)	Receptor	431	Índia	Vivo	43,18	33,87	Múltipla	NR	9	6
Meier-KriescheH et al^22^ (2001)	Receptor	73.477	EUA	Ambos	NR^a^	NR^a^	Múltipla	1988 a 1997	NR	8
Inoue et al^14^ (2002)	Receptor e doador-receptor	205	Japão	Ambos	NR^a^	NR^a^	Cisclosporina ou FK506	1987 a 2000	NR	7
Moosa^23^ (2003)	Receptor	542	África do Sul	Cadáver	NR^a^	37	Múltipla	1976 a 1999	75,6	7
Chen et al^6^ (2013)	Receptor	766	China	Ambos	NR^a^	NR^a^	Múltipla	1988 a 2009	NR	7
Ellison etal^8^ (1994)	Doador-receptor	3.314	EUA	Ambos	NR	NR	NR	1987 a 1992	NR	8
Shaheen et al^38^ (1998)^b^	Doador-receptor	406	Arábia Saudita	Vivo	31,3	34,3	Ciclosporina	NR	55,2	7
Vereerstraeten et al^42^ (1999)	Doador-receptor	741	Bélgica	Cadáver	34,8	36,9	Múltipla	1983 a 1997	NR	7
Zeier et al^43^ (2002)	Doador-receptor	119.195	49 países	Ambos	38,3	44,3	NR	1985 a 2000	NR	8
Kayler et al^16^ (2003)	Doador-receptor	30.258	EUA	Vivo	NR^a^	NR^a^	NR	1990 a 1999	NR	8
Kwon e Kwak^19^ (2004)	Doador-receptor	614	Coreia do Sul	Vivo	NR^a^	NR^a^	NR	1979 a 2002	NR	7
Pugliese et al^34^ (2005)	Doador-receptor	3.233	Itália	Cadáver	42,1	44,5	NR	1995 a 2000.	NR	8
Jacobs et al^15^ (2007)	Doador-receptor	730	EUA	Vivo	39,7	46,4	NR	1979 a 1994	NR	8
Gratwohl et al^12^ (2008)	Doador-receptor	195.516	45 países	Cadáver	38	44,7	NR	1985 a 2004	67,2	8
Kim e Gill^17^ (2009)	Doador-receptor	117.877	EUA	Cadáver	NR^a^	NR^a^	NR	1990 a 2004	NR^a^	7
Lankarani et al^20^ (2009)	Doador-receptor	2.649	Iran	Vivo	NR^a^	NR^a^	NR	1992 a 2005	NR	8
Shaheen et al^39^ (2010)	Doador-receptor	524	Arábia Saudita	Cadáver	33,6	33,9	Múltipla	2003 a 2007	20,9	8
Głyda et al^11^ (2011)	Doador-receptor	154	Polônia	Cadáver	NR^a^	NR^a^	Múltipla	NR	NR	7
Zukowski et al^45^ (2011)	Doador-receptor	230	Polônia	Cadáver	33,1	37,6	NR	NR	NR	8
Abou-Jaoude et al^1^ (2012)	Doador-receptor	135	Líbano	Ambos	NR^a^	NR^a^	Múltipla	1998 a 2007	NR	7
Tan et al^40^ (2012)	Doador-receptor	188.507	EUA	Ambos	NR^a^	NR^a^	NR	1988 a 2006	NR	8

NR: não relata; NR^a^: não relata da forma desejada.

^b^ Coorte prospectiva.

Para todas as comparações entre gênero, a maioria dos participantes dos estudos (doadores, receptores e doadores-receptores) era do sexo masculino. Todos os estudos incluídos avaliaram sobrevida do enxerto, mas apenas oito avaliaram sobrevida do paciente.^1^ ^,^ ^14^ ^,^ ^20^ ^,^ ^22^ ^,^ ^23^ ^,^ ^27^ ^,^ ^39^ ^,^ ^43^

Dos 29 estudos incluídos, dois obtiveram a pontuação seis na escala de New-Castle Ottawa;^2^ ^,^ ^4^ 14 (48,3%) obtiveram sete; e 13, oito (Tabela 2).

Dos seis estudos incluídos na revisão sistemática para a comparação de gênero do doador, cinco foram incluídos na metanálise para o desfecho sobrevida do enxerto.^3^ ^-^ ^5^ ^,^ ^29^ ^,^ ^41^ O estudo de Neugarten et al^25^ não apresentou dados numéricos suficientes para a análise quantitativa. Os estudos incluídos na comparação gênero do doador não avaliaram sobrevida do paciente.

Para a sobrevida do enxerto, os riscos relativos (RR) agrupados em ordem por tempo de acompanhamento de um, dois, três, cinco, oito e 10 anos foram, respectivamente, 1,02 (IC95% 0,97;1,07; p = 0,43; I^2^ = 70,0%), 1,03 (IC95% 1,00;1,07; p = 0,07; I^2^ = 27,0%), 1,02 (IC95% 0,94;1,12; p = 0,58; I^2^ = 67,0%), 0,89 (IC95% 0,79;1,00; p = 0,06; I^2^ = 0%), 0,86 (IC95% 0,73;1,02; p = 0,09; I^2^ = 11,0%;) e 0,82 (IC95% 0,68;0,98; p = 0,03; I^2^ = 0%). Apenas aos 10 anos de acompanhamento a diferença foi significativa para sobrevida do enxerto, favorecendo doadores do gênero masculino. A heterogeneidade foi alta e significante para os tempos de acompanhamento de um e três anos (Tabela 3).

**Tabela 3:** Resumo das metanálises para sobrevida do enxerto segundo o gênero do doador (DF ou DM) e do receptor (RF ou RM); e sobrevida do paciente segundo o gênero do receptor de transplante de renal.

Desfecho/tempo	Estudos	Participantes	RR	IC95%	p^a^	I^2^ (%)^b^
Sobrevida do enxerto	DF *versus* DM					
1 ano	^3,5,30^	7.478	1,02	0,97;1,07	0,43	70,0^c^
2 anos	^5,30,41^	8.154	1,03	1,00;1,07	0,07	27,0
3 anos	^5,30,41^	8.154	1,02	0,94;1,12	0,58	67,0^c^
5 anos	^3,30^	589	0,89	0,79;1,00	0,06	0
8 anos	^4,30^	761	0,86	0,73;1,02	0,09	11,0
10 anos	^3,30^	589	0,82	0,68;0,98	0,03	0
Sobrevida do enxerto	RF *versus* RM					
1 ano	^2,5,10^	8.348	1,01	0,99;1,03	0,30	0
5 anos	^2,6,15^	1.402	0,99	0,88;1,12	0,88	75,0^c^
10 anos e mais	^15,24^	747	1,23	0,68;2,24	0,50	95,0^c^
Sobrevida do paciente	RF *versus* RM					
10 anos e mais	^15,24^	747	0,96	0,67;1,37	0,81	95,0^c^

RR: risco relativo

^a^ Valor de p < 0,10 do teste Z para todos os efeitos.

^b^ Valor de I^2^ > 40,0% indica heterogeneidade estatística entre os estudos.

^c^ Heterogeneidade significante (p < 0,10).

Dos oito estudos incluídos na revisão sistemática para a comparação de gênero do receptor, seis foram incluídos na metanálise para o desfecho sobrevida do enxerto.^2^ ^,^ ^5^ ^,^ ^6^ ^,^ ^14^ ^,^ ^23^ ^,^ ^35^ Os estudos de Nyberg et al^27^ e Meier-Kriesche et al^22^ não apresentaram dados numéricos para serem incluídos na análise quantitativa. Assim, as metanálises foram realizadas nos tempos de seguimento (um; cinco; e 10 ou mais anos).

Na metanálise referente à sobrevida do enxerto para um ano foram incluídas quatro comparações de três estudos. No estudo de Sánchez Garcia et al,^35^ (1989) comparou-se a influência da terapia imunossupressora sobre o gênero, dividido em dois grupos: Sánchez Garcia et al^35^ (1989a), tratados com ciclosporina, e Sánchez Garcia et al^35^ (1989b), não tratados com ciclosporina.

Para a sobrevida do enxerto, os RR agrupados em ordem por tempo de acompanhamento de um, cinco, 10 anos e mais foram, respectivamente, 1,01 (IC95% 0,99;1,03; p = 0,30; I^2^ = 0%), 0,99 (IC95% 0,88;1,12; p = 0,88; I^2^ = 75,0%), 1,23 (IC95% 0,68;2,24; p = 0,50; I^2^ = 95,0%). Não houve diferença significante em nenhum dos tempos de acompanhamento, sem favorecimento para o gênero de receptor entre os grupos. A heterogeneidade foi alta e significante para cinco e 10 ou mais anos de acompanhamento (Tabela 3).

Para a sobrevida do paciente foram incluídos na metanálise dois estudos.^14^ ^,^ ^24^ A metanálise foi realizada para o tempo de acompanhamento de 10 anos ou mais, a heterogeneidade foi alta e a diferença não foi significativa (RR = 0,96; IC95% 0,67;1,37; p = 0,81; I^2^ = 95,0%) (Tabela 3).

Para avaliar gênero do doador-receptor, foram analisadas seis comparações: DM-RM *versus* DF-RM, DM-RM *versus* DF-RF, DM-RM *versus* DM-RF, DM-RF *versus* DF-RF, DM-RF *versus* DF-RM, DF-RF *versus* DF-RM. Para o desfecho sobrevida do enxerto, foram incluídos 13 estudos^1^ ^,^ ^5^ ^,^ ^8^ ^,^ ^11^ ^,^ ^15^ ^-^ ^17^ ^,^ ^19^ ^,^ ^20^ ^,^ ^38^ ^-^ ^40^ ^,^ ^43^ nas metanálises (Tabela 4).

**Tabela 4:** Resumo das metanálises para sobrevida do enxerto segundo o gênero do doador-receptor de transplante renal.

Características gerais		DM-RM *versus* DF-RM					DM-RM *versus* DF-RF				
Tempo	Estudos	N^o^ pacientes	RR	IC95%	p^a^	I^2^ (%)^b^	N° pacientes	RR	IC95%	p^a^	I^2^ (%)^b^
1 ano	^1,5,8,15,16,17,20,39,40^	211.025	1,03	1,01;1,05	< 0,01	92,0^c^	181.223	1,02	1,01, 1,04	< 0,01	83,0^c^
2 anos	^5,8,15,20^	8.411	1,05	1,03;1,08	<0,01	25,0	7.575	1,02	1,00;1,04	0,10	9,0
3 anos	^5,8,15,17,20,39^	79.979	1,06	1,02;1,09	< 0,01	72,0^c^	69.993	1,05	1,01;1,09	0,02	73,0^c^
5 anos	^11,16,17,19,20,38,40^	204.668	1,15	1,00;1,33	0,05	100^c^	175.602	1,02	1,01;1,03	< 0,01	50,0^c^
10 anos	^17,40,43^	258.63	1,08	1,05;1,11	< 0,01	91,0^c^	223.404	1,02	0,97;1,07	0,43	97,0^c^

Características gerais		DM-RM *versus* DM-RF					DM-RF *versus* DF-RF				

Tempo	Estudos	N^o^ pacientes	RR	IC95%^a^	p^b^	I^2^ (%)^c^	N° pacientes	RR	IC95%^a^	p^b^	I^2^ (%)^c^

1 ano	^1,5,8,15,16,17,20,39,40^	196.191	1,01	1,01;1,02	< 0,01	67,0^c^	139.859	1,01	0,99;1,02	0,30	77,0^c^
2 anos	^5,8,15,20^	9.086	1,01	0,99;1,03	0,32	0	5.171	1,01	0,98;1,03	0,61	0
3 anos	^5,8,15,17,20,39^	80.495	1,03	1,00;1,06	0,07	76,0^c^	51.994	1,01	1,00;1,02	< 0,01	0
5 anos	^11,16,17,19,20,38,40^	189.508	1,00	0,97;1,03	0,98	91,0^c^	135.798	1,01	1,00;1,03	0,11	66,0^c^
10 anos	^17,40,43^	246.909	0,96	0,86;1,06	0,38	99,0^c^	166.817	1,04	1,02;1,06	< 0,01	80,0^c^

Características gerais		DM-RF *versus* DF-RM					DF-RF *versus* DF-RM				

Tempo	Estudos	N^o^ pacientes	RR	IC95%^a^	p^b^	I^2^ (%)^c^	N° pacientes	RR	IC95%^a^	p^b^	I^2^ (%)^c^

1 ano	^1,5,8,15,16,17,20,39,40^	169.666	1,01	1,00;1,03	0,15	90,0^c^	154.693	1,01	0,99;1,02	0,45	80,0^c^
2 anos	^5,8,15,20^	5.679	1,05	1,01;1,10	0,01	36,0	4.496	1,03	0,99;1,07	0,18	51,0^c^
3 anos	^5,8,15,17,20,39^	61.980	1,03	1,00;1,05	0,04	44,0^c^	51.478	1,01	0,98;1,04	0,53	44,0
5 anos	^11,16,17,19,20,38,40^	164.864	1,18	1,03;1,35	0,02	100^c^	150.958	1,14	0,99;1,31	0,07	100^c^
10 anos	^17,40,43^	202.176	1,10	1,01;1,21	0,03	99,0^c^	176.671	1,06	0,98;1,14	0,14	98,0^c^

RR: risco relativo

^a^ Valor de p < 0,10 do teste Z para todos os efeitos.

^b^ Valor de I^2^ > 40,0% indica heterogeneidade estatística entre os estudos.

^c^ Heterogeneidade significante (p < 0,10).

Os outros estudos não apresentaram dados suficientes para a análise quantitativa.^3^ ^,^ ^10^ ^,^ ^12^ ^,^ ^14^ ^,^ ^25^ ^,^ ^42^ ^,^ ^45^ Para o desfecho sobrevida do paciente, dois estudos foram incluídos na metanálise.^1^ ^,^ ^20^

Os estudos de Ellison et al^8^ e Tan et al^40^ avaliaram transplantes de doadores vivos e falecidos, separadamente, sendo incluída na metanálise a somatória do número de eventos e de participantes de cada estudo considerando doadores vivos e falecidos. No estudo de Abou-Jaoude et al^1^ foi avaliada a sobrevida do enxerto geral e a sobrevida do enxerto censurada pela morte com enxerto funcionante, sendo utilizada na metanálise a sobrevida sem censura.

Na comparação DM-RM *versus* DF-RM, os RR agrupados para a sobrevida do enxerto em ordem por tempo de acompanhamento de um, dois, três, cinco e 10 anos foram, respectivamente, 1,03 (IC95% 1,01;1,05; p = 0,0002; I^2^ = 92,0%), 1,05 (IC95% 1,03;1,08; p < 0,0001; I^2^ = 25,0%), 1,06 (IC95% 1,02;1,09; p = 0,0008; I^2^ = 72,0%), 1,15 (IC95% 1,00;1,33; p = 0,05; I^2^ = 100%) e 1,08 (IC95% 1,05;1,11; p < 0,00001; I^2^ = 91,0%). A taxa de sobrevida do enxerto foi significativamente maior em todos os tempos de acompanhamento avaliados, favorecendo o par DM-RM. A heterogeneidade foi alta e significante para todos os períodos, exceto aos dois anos de acompanhamento. A análise de sobrevida do paciente foi realizada apenas para o tempo de acompanhamento de um ano e não houve favorecimento de nenhum par (RR = 0,99; IC95% 0,96;1,02; p = 0,52; I^2^ = 0,0%).

Na comparação DM-RM *versus* DF-RF, os RR agrupados para a sobrevida do enxerto em ordem por tempo de acompanhamento de um, dois, três, cinco e 10 anos foram, respectivamente, 1,02 (IC95% 1,01;1,04; p = 0,0008; I^2^ = 83,0%), 1,02 (IC95% 1,00;1,04; p < 0,10; I^2^ = 9,0%), 1,05 (IC95% 1,01;1,09; p = 0,02; I^2^ = 73,0%), 1,02 (IC95% 1,01;1,03; p = 0,0004; I^2^ = 50,0%) e 1,02 (IC95% 0,97;1,07; p = 0,43; I^2^ = 97,0%). A taxa de sobrevida do enxerto foi significativamente maior nos tempos de acompanhamento de um, três e cinco anos, favorecendo o par DM-RM. A heterogeneidade não foi significante apenas para dois anos de acompanhamento. A metanálise de sobrevida do paciente não mostrou favorecimento para nenhum par (RR = 0,98; IC95% 0,95;1,01; p = 0,21; I^2^ = 0%).

Na comparação DM-RM *versus* DM-RF, os RR agrupados para a sobrevida do enxerto, em ordem por tempo de acompanhamento de um, dois, três, cinco e 10 anos foram, respectivamente, 1,01 (IC95% 1,01;1,02; p = 0,0009; I^2^ = 67,0%), 1,01 (IC95% 0,99;1,03; p = 0,32; I^2^ = 0%), 1,03 (IC95%1,00;1,06; p = 0,07; I^2^ = 76,0%), 1,00 (IC95% 0,97;1,03; p = 0,98; I^2^ = 91,0%) e 0,96 (IC95% 0,86;1,06; p = 0,38; I^2^ = 99,0%). A taxa de sobrevida do enxerto foi significativamente maior apenas no tempo de acompanhamento de um ano, favorecendo o par DM-RM. A heterogeneidade não foi significante apenas para dois anos de acompanhamento. A metanálise de sobrevida do paciente não mostrou favorecimento para nenhum par (RR = 1,00; IC95% 0,98;1,03; p = 0,86; I^2^ = 0%).

Na comparação DM-RF *versus* DF-RF, os RR agrupados para a sobrevida do enxerto, em ordem por tempo de acompanhamento de um, dois, três, cinco e 10 anos foram, respectivamente, 1,01 (IC95% 0,99;1,02; p = 0,30; I^2^ = 77,0%), 1,01 (IC95% 0,98;1,03; p = 0,61; I^2^ = 0%), 1,01 (IC95% 1,00;1,02; p = 0,0007; I^2^ = 0%), 1,01 (IC95% 1,00;1,03; p = 0,11; I^2^ = 66,0%) e 1,04 (IC95% 1,02;1,06; p = 0,0003; I^2^ = 80,0%). A taxa de sobrevida do enxerto foi significativamente maior nos tempos de acompanhamento de três e 10 anos, favorecendo o par DM-RF. A heterogeneidade não foi significante apenas para dois e três anos de acompanhamento. A metanálise de sobrevida do paciente não mostrou favorecimento para nenhum par (RR = 0,98; IC95% 0,94;1,01; p = 0,19; I^2^ = 0%).

Na comparação DM-RF *versus* DF-RM, os RR agrupados para a sobrevida do enxerto em ordem por tempo de acompanhamento de um, dois, três, cinco e 10 anos foram, respectivamente, 1,01 (IC95% 1,00;1,03; p = 0,15; I^2^ = 90,0%), 1,05 (IC95% 1,01;1,10; p = 0,01; I^2^ = 36,0%), 1,03 (IC95% 1,00;1,05; p = 0,04; I^2^ = 44,0%), 1,18 (IC95% 1,03;1,35; p = 0,02; I^2^ = 100%) e 1,10 (IC95% 1,01;1,21; p = 0,03; I^2^ = 99,0%). A taxa de sobrevida do enxerto foi significativamente maior em todos os tempos de seguimento, exceto em um ano, favorecendo o par DM-RF. A heterogeneidade foi alta e significante para todos os períodos, exceto aos dois anos de acompanhamento. A metanálise de sobrevida do paciente não mostrou favorecimento para nenhum par (RR = 0,99; IC95% 0,95;1,02; p = 0,44; I^2^ = 0%).

Na comparação DF-RF *versus* DF-RM, os RR agrupados para a sobrevida do enxerto em ordem por tempo de acompanhamento de um, dois, três, cinco e 10 anos foram, respectivamente, 1,01 (IC95% 0,99;1,02; p = 0,45; I^2^ = 80,0%), 1,03 (IC95% 0,99;1,07; p = 0,18; I^2^ = 51,0%), 1,01 (IC95% 0,98;1,04; p = 0,53; I^2^ = 44,0%), 1,14 (IC95% 0,99;1,31; p = 0,07; I^2^ = 100%) e 1,06 (IC95% 0,98;1,14; p = 0,14; I^2^ = 98,0%). Não houve diferença significante na taxa de sobrevida do enxerto nos tempos de acompanhamento. A heterogeneidade foi alta e significante para todos os períodos, exceto aos três anos de acompanhamento. A metanálise de sobrevida do paciente não mostrou favorecimento para nenhum par (RR = 1,01; IC95% 0,97;1,06; p = 0,54; I^2^ = 0%).

## DISCUSSÃO

Esta metanálise avaliou a influência do gênero de doadores, receptores e combinação doador-receptor na sobrevida do enxerto e do paciente no transplante renal.

Para a comparação entre gênero de doador, foram avaliados 9.022 pacientes na metanálise. O gênero do doador não favoreceu a sobrevida do enxerto na avaliação de um, dois, três, cinco e oito anos de acompanhamento (p > 0,05). Apenas no tempo de acompanhamento de 10 anos houve diferença significativa favorecendo os doadores do gênero masculino (p = 0,03). O estudo de Muller^24^ concluiu que enxertos renais de pacientes do gênero masculino funcionam melhor que aqueles de pacientes do gênero feminino, em longo prazo. Vários estudos sugerem que, nesses casos, os enxertos de doadoras são mais antigênicos, o que pode explicar a menor sobrevida.^22^ ^,^ ^30^ ^,^ ^31^ ^,^ ^36^

Para a comparação entre gênero de receptor, em todos os períodos de acompanhamento, foram avaliados 9.593 pacientes na metanálise e não foi observada diferença significativa na sobrevida do enxerto. Na análise de sobrevida do paciente foram avaliados 747 pacientes e não foram encontradas diferenças significativas entre os estudos. O estudo de Busson e Benoit^5^ avaliou a influência do gênero entre receptores, doadores e da combinação doadores-receptores e não encontrou diferenças significativas apenas para o gênero do receptor.

Na comparação entre gênero de doador-receptor, participaram 471.252 pacientes. Evidencia-se que no par DM-RM houve melhores resultados em todas as comparações (DM-RM *versus* DF-RM, DM-RM *versus* DF-RF, DM-RM *versus* DM-RF), ou seja, os transplantes de rins de doadores masculinos em receptores masculinos apresentaram melhores taxas para sobrevida do enxerto. Entretanto, no par DF-RM houve os piores resultados (DF-RM *versus* DM-RM, DF-RM *versus* DM-RF), exceto na comparação DF-RM *versus* DF-RF. Esses resultados são comparáveis com outras revisões.^7^ ^,^ ^44^

A avaliação das diferenças entre gênero é importante para melhorar os resultados no transplante. Homens e mulheres possuem fatores biologicamente desiguais, bem como condições diferentes do corpo, circunstâncias hormonais e respostas imunes, além de demandas metabólicas e funcionais, que podem influenciar nos resultados do transplante renal.^44^ Estudos discutem que a incompatibilidade entre gênero no par DF-RM influencia negativamente a sobrevida do enxerto devido ao volume do rim e número de néfrons, sendo importante a relação do peso do enxerto com o peso do destinatário.^26^ ^,^ ^33^

Giral et al^10^ analisaram as consequências da redução da massa renal em resultados após transplante renal e concluíram que a massa do enxerto renal impacta a taxa de filtração glomerular e proteinúria. Os autores sugerem evitar grande inadequação do peso do rim e do destinatário, uma vez que isso pode influenciar significativamente na função do transplante em longo prazo.

Esta metanálise incluiu somente estudos de coorte. Uma das limitações de revisões sistemáticas com metanálise de estudos observacionais refere-se ao viés de seleção intrínseco a esse delineamento de estudo e a fatores de confusão não controlados. Estudos observacionais de coorte são estudos realizados no mundo real em condições não controladas. Com isso, foram observadas diferenças no número de participantes entre os grupos, tipo de doadores (vivos, falecidos ou ambos), número de transplantes, período de seguimento, entre outros. Apesar disso, os estudos observacionais têm a vantagem de reunir um número grande de pacientes e melhor representar o mundo real.

Outra limitação na interpretação dos resultados foi a heterogeneidade estatística entre os estudos encontrada nas metanálises. O pequeno número de estudos incluídos nas comparações e a falta de informações completas e precisas nos estudos dificultaram a explicação para as fontes de heterogeneidade. A maioria dos estudos não apresentou a terapia imunossupressora, idade (doador e receptor) e tempo de seguimento. A análise de sensibilidade com inclusão e exclusão de estudos em cada comparação, no geral, não alterou a direção dos desfechos, com alterações pouco relevantes no valor da heterogeneidade, além de não fornecer informação sobre as possíveis causas para a heterogeneidade.

Entretanto, os resultados desta revisão podem ser considerados para a tomada de decisão na prática clínica pelas equipes transplantadoras, uma vez que representa o melhor nível de evidência sobre o assunto.

A incompatibilidade entre gênero deve ser evitada sempre que possível. O gênero deve ser considerado como um critério na escolha da alocação de órgãos dos doadores e receptores, sendo que a combinação de gênero pode fazer a diferença nas taxas de sobrevida. Entretanto, essa é uma realidade comprometida na prática clínica, devido à escassez de doadores e ao aumento do número de pacientes na lista de espera para transplante, tornando-se necessária uma mudança nesse cenário a fim de melhorar a alocação dos órgãos.

Em conclusão, gênero do receptor e do doador avaliado isoladamente não influencia a sobrevida do enxerto e do paciente. Contudo, a combinação gênero doador-receptor pode ser um fator determinante para a sobrevida do enxerto, favorecendo o par DM-RM.

## Footnotes

a Higgins JPT, Green S, editors. Cochrane handbook for systematic reviews of interventions: version 5.1.0. London: The Cochrane Collaboration; 2011 [citado 2014 fev 6]. Disponível em: http://www.cochrane-handbook.org

b Wells GA, Shea B, O’Connelll D, Peterson J, Welch V, Losos M, et al. The Newcastle-Ottawa Scale (NOS) for assessing the quality of nonrandomised studies in meta-analyses. Ottawa: Ottawa Hospital Research Institute; 2014 [citado 2014 fev 6]. Disponível em: http://www.ohri.ca/programs/clinical_epidemiology/oxford.asp
